# Exploring factors associated with personal recovery in bipolar disorder

**DOI:** 10.1111/papt.12339

**Published:** 2021-03-19

**Authors:** Jannis T. Kraiss, Peter M. ten Klooster, Emily Frye, Ralph W. Kupka, Ernst T. Bohlmeijer

**Affiliations:** ^1^ Department of Psychology, Health, and Technology Center for eHealth and Well‐being Research University of Twente Enschede Netherlands; ^2^ Psychiatry Amsterdam Public Health Research Institute Vrije Universiteit Amsterdam UMC The Netherlands

**Keywords:** association, bipolar disorder, emotion regulation, recovery, social participation

## Abstract

**Background:**

Personal recovery is increasingly recognized as important outcome for people with bipolar disorder (BD), but research addressing associated factors of personal recovery in this group remains scarce. This study aimed to explore the association of sociodemographic variables, social participation, psychopathology, and positive emotion regulation with personal recovery in BD.

**Methods:**

Baseline data from a randomized controlled trial and survey data were combined (*N = *209) and split into a training (*n* = 149) and test sample (*n* = 60). Block‐wise regression analyses and model training were used to determine the most relevant predictors. The final parsimonious model was cross‐validated in the test sample.

**Results:**

In the final parsimonious model, satisfaction with social roles (β = .442, *p* < .001), anxiety symptoms (β = −.328, *p* < .001), manic symptoms (β = .276, *p* < .001), and emotion‐focused positive rumination (β = .258, *p* < .001) were independently associated with personal recovery. The model explained 57.3% variance in personal recovery (adjusted *R* ^2^ = .561) and performed well in predicting personal recovery in the independent test sample (adjusted *R* ^2^ = .491).

**Conclusions:**

Our findings suggest that especially social participation, anxiety and positive rumination might be relevant treatment targets when aiming to improve personal recovery.

**Practitioner points:**

- Personal recovery is considered an increasingly important outcome for people with chronic mental health conditions, including bipolar disorder.
- We found that anxiety and manic symptoms as well as positive rumination and social participation were independently associated with personal recovery in bipolar disorder.
- Therefore, these outcomes might be relevant treatment targets when aiming to improve personal recovery in bipolar disorder.
- Possible interventions to improve these outcomes are discussed, including supported employment and vocational rehabilitation for social participation and exercising with savoring strategies to increase positive rumination.

## Background

Bipolar disorder (BD) is a chronic mental disorder characterized by recurrent depressive and (hypo)manic mood episodes, intertwined with euthymic phases, in which patients are relatively symptom‐free. A distinction is made between bipolar I (BDI) and bipolar II (BDII) disorder. In BDII, an individual experiences depressive and hypomanic episodes, but never a full‐blown manic episode (Goodwin & Jamison, 2007). BD carries the highest suicide risk compared to all other psychiatric illnesses (Miller & Black, 2020; Plans et al., 2019) and is associated with impairments in cognitive functioning (Cotrena, Branco, Shansis, & Fonseca, 2016), quality of life (Sylvia et al., 2017), high caregiver burden (Blanthorn‐Hazell, Gracia, Roberts, Boldeanu, & Judge, 2018), and substantial societal costs (Cloutier, Greene, Guerin, Touya, & Wu, 2018; Kraiss, Wijnen, Kupka, Bohlmeijer, & Lokkerbol, 2020).

Besides clinical and functional recovery, the importance of improving personal recovery in people with mental disorders is increasingly recognized. Personal recovery has been defined as ‘a deeply personal, unique process of changing one’s attitudes, values, feelings, goals skills and/or roles […] and a way of living a satisfying, hopeful, and contributing life, even with limitations caused by the illness’ (Anthony, 1993, p. 527). Especially anglophone countries emphasize the importance of recovery‐focused approaches in their policies and health services (Bird et al., 2014). A literature review identified five key components of personal recovery in mental disorders, namely connectedness, hope, identity, meaning, and empowerment. The authors of this review also concluded that more research is needed about contributing factors for personal recovery (Leamy, Bird, Le Boutillier, Williams, & Slade, 2011). Personal recovery‐related outcomes are highly valued by people with mental disorders and are considered important objectives of treatment (Leamy et al., 2011; Mead & Copeland, 2000; de Vos et al., 2017), also increasingly for people with BD (Jones et al., 2015). People with BD describe similar factors as relevant for personal recovery. Respect, hope and empowerment (Tse et al., 2014), becoming the director of you own life (Warwick, Tai, & Mansell, 2019) and social support and companionship (Mansell, Powell, Pedley, Thomas, & Jones, 2010) were described as important parts of recovery. Also, research suggests that recovery in BD is not only about being symptom‐free (Todd, Jones, & Lobban, 2012) and that clinical recovery alone may misrepresent treatment success from the perspective of people with BD (Mezes, Lobban, Costain, Longson, & Jones, 2020). However, quantitative research about factors that are associated with personal recovery in BD remains scarce.

One factor that might be an important determinant of personal recovery from chronic psychiatric conditions is social role participation (Jaeger & Hoff, 2012), referring to the degree to which an individual is able to fulfil social roles, such as having intimate relationships or being a working employee (Oude Voshaar et al., 2016). Social functioning is described as integral part of recovery (Liberman, 2009). Having less satisfaction with social roles has been associated with increased depression and stress, and greater role limitations were associated with increased health care utilization (Gignac et al., 2013). Also, the degree to which an individual is able to fulfil social roles has been shown to be important for building and maintaining self‐esteem (Gordeev et al., 2010). Although the direct link between personal recovery and social participation has not yet been investigated in BD, increased health care utilization and depression symptoms as well as lower self‐esteem might be indicative of low recovery. Furthermore, being able to work has been described as relevant factor for recovery by people with BD (Borg, Veseth, Binder, & Topor, 2013). Therefore, social role participation might also be a relevant determinant for personal recovery in BD.

Another important process for the onset, persistence, and recurrence of mental disorders is positive emotion regulation (Carl, Soskin, Kerns, & Barlow, 2013), which is defined as the way in which people react to their positive affective states. Two important positive emotion regulation strategies are *dampening* and *positive rumination*. Dampening is described as the suppression of positive moods to reduce the intensity of positive affect (Quoidbach, Berry, Hansenne, & Mikolajczak, 2010), while positive rumination has been described as the tendency to respond to positive emotions with recurrent thoughts about positive experiences (Feldman, Joormann, & Johnson, 2008).

Research suggests that dampening might be a particular maladaptive strategy in BD (Gilbert, Nolen‐Hoeksema, & Gruber, 2013). Dampening has been shown to be heightened among people with BD (Edge et al., 2013; Johnson, Tharp, Peckham, & McMaster, 2016) and to predict depressive and also manic symptoms over the course of 6 months in remitted persons with BD (Gilbert et al., 2013). Mansell (2016) describes the fear of becoming manic and experiencing relapse as one of the greatest problems in BD. One consequence of this fear might be dampening of positive emotions and avoiding positive experiences (Edge et al., 2013), which may prevent people from leading a meaningful life and hinder recovery. Dampening could thus eventually be an obstacle for personal recovery in BD. Dampening is negatively associated with quality of life (Edge et al., 2013) and positively associated with depressive symptoms in BD (Gilbert et al., 2013). Both decreased quality of life and increased depressive symptoms might be an indication for decreased recovery and dampening might thus also be a relevant determinant of personal recovery in BD. Positive rumination has been shown to be positively related to higher lifetime frequency of mania (Gruber, Eidelman, Johnson, Smith, & Harvey, 2011) and lifetime diagnoses of mania or hypomania (Johnson, McKenzie, & McMurrich, 2008). Interestingly, research shows that extreme appraisal of positive emotions is related to worsened illness course in BD (Ford, Mauss, & Gruber, 2015), suggesting that positive rumination might be a maladaptive strategy in BD. Conversely, research also shows that increased positive emotions are associated with better functioning in BD (Johnson et al., 2016). Considering that positive rumination has been linked to increased positive emotions (Quoidbach et al., 2010), positive rumination, depending on the level of rumination, might thus also be an adaptive strategy that has the potential to improve functioning. This makes positive rumination an interesting candidate to examine as potential determinant of personal recovery in BD.

Although personal recovery is an important outcome for people with severe mental disorders, little is known about factors contributing to personal recovery in people with BD. One recent study explored the role of negative beliefs about mood swings and self‐referent appraisals of mood‐related experiences as correlates of personal recovery in BD, but social role participation or positive emotion regulation was not included in their study (Dodd, Mezes, Lobban, & Jones, 2017). However, previous research suggests that positive emotion regulation and social role participation might be important correlates of personal recovery. To our knowledge, the actual association of these processes with personal recovery in BD remains unknown. Also, it has not yet been investigated whether these processes are associated with personal recovery above and beyond symptomatology. Furthermore, little is known about the role of sociodemographic factors for personal recovery in BD, but there is evidence suggesting that for example active work status is beneficial for people with BD (Dodd et al., 2017).

Increasing our knowledge about factors associated with personal recovery in BD will inform clinical practice and help to enhance recovery‐focused therapies, which may eventually lead to more effective treatments and improve prognoses for individuals with BD. The current study aims to widen the body of knowledge in this field by exploring an array of sociodemographic variables, as well as social participation, positive emotion regulation and psychopathology as potential correlates of personal recovery in BD. In the present study, personal recovery is operationalized as a generic and comprehensive construct, including all five dimensions from the evidence‐based CHIME‐framework (Leamy et al., 2011). Based on prior research, we hypothesized that social role participation will be positively associated with personal recovery. Further, we expected that positive rumination will be positively associated with personal recovery and that overall symptomatology and dampening show a negative relationship with personal recovery.

## Method

### Procedure

Data from two studies were combined for the current study. The first dataset (*n* = 119) comes from an online cross‐sectional survey study conducted between April and July 2018 (Kraiss, ten Klooster, et al., 2019). The second dataset (*n* = 90) was collected between September 2018 and March 2020 for the baseline assessment of a randomized controlled trial (RCT) on the effectiveness of a psychological intervention for patients with BD (Kraiss et al., 2018). The Ethics Committee of the corresponding University approved the first study. The second study has been approved by a Medical Ethical Research Committee. Merging both datasets for the current study was not originally planned, but decided later in the process of the RCT study, since both studies include similar outcome measures and this way the statistical power could be increased.

For the survey study, adult participants were gathered through convenience sampling via the Dutch patient association for people with bipolar disorder. Diagnoses were based on self‐report and not confirmed by a clinical interview. For the RCT, participants had to sign an informed consent before participation. The most relevant inclusion criteria for the RCT were as follows: (1) diagnosis of BDI or BDII, (2) ages 18–65 and (3) currently not in an acute depressive or (hypo)manic episode. A detailed overview of the procedure and in‐ and exclusion criteria of the RCT can be found in the study protocol (Kraiss et al., 2018).

### Measures

Participants were asked to provide demographical features, including age, gender, relationship status and education. Participants were asked whether they took medication in the context of BD and whether they had been admitted to a psychiatric hospital because of their BD in the past months. The following constructs were assessed:

*Personal recovery* was assessed with the 15‐item Questionnaire about the Process of Recovery (Neil et al., 2009). Respondents were asked how they felt in the past 7 days (e.g. ‘I feel better about myself’) on a 5‐point Likert scale ranging from 0 (*strongly disagree*) to 4 (*strongly agree*), with higher scores being indicative of more personal recovery. The range of possible total QPR scores ranges from 0 to 60. Although a specific measure of personal recovery for BD has been developed, the Bipolar Recovery Questionnaire (BRQ; Jones, Mulligan, Higginson, Dunn, & Morrison, 2013), we chose the QPR in our studies because it represents a less burdensome and more feasible alternative to the 36‐item BRQ and fits well with the evidence‐based generic CHIME‐framework (Leamy et al., 2011), since every item of the QPR maps to one of its dimensions (Shanks et al., 2013). The Dutch version of the QPR has been translated and validated in patients with BD (Kraiss, Ten Klooster, et al., 2019). Cronbach’s alpha in the current (combined) sample was 0.93.
*Social role participation* was measured with the Short‐Social Role Participation Questionnaire (S‐SRPQ; Oude Voshaar et al., 2016) containing the two subscales satisfaction with role performance (six items) and experienced difficulties with role performance (six items). The S‐SRPQ asks participants to specify their satisfaction and experienced difficulties in relation to six different social situations (e.g. work, relationships) on a 5‐point Likert scale ranging from 1 (not satisfied at all / no difficulties at all) to 5 (very much satisfied / not possible). Higher scores on the corresponding subscales are indicative of more satisfaction with role performance or more experienced difficulties with role performance, respectively. In the current study, we used mean scores for both subscales, as this is the usual way of scoring the S‐SRPQ. Therefore, the scores can range from 1 to 5 in the current study. Cronbach’s alpha in the current sample was .73 for the subscale satisfaction with social role performance and .77 for the subscale difficulties with social roles.
*Anxiety symptoms* were assessed with the anxiety subscale of the Hospital Anxiety and Depression Scale (HADS‐A; Spinhoven et al., 1997; Zigmond & Snaith, 1983). The HADS‐A measures the presence of mild forms of anxiety with 7 items. The presence of symptoms in the past week is rated on a scale from 0 (not at all) to 3 (very often). The scores of the HADS‐A can range from 0 to 21. Higher scores are indicative of increased anxiety. Cronbach’s alpha in the current study was .86 for the anxiety subscale.
*Manic symptoms* were assessed using 5‐item Altman Self‐Rating Mania Scale (ASRM; Altman, Hedeker, Peterson, & Davis, 1997), which consists of statements representing manic symptoms, such as inflated self‐confidence or increased chattiness. Items are rated on a 5‐point Likert scale containing different answering categories. The scores of the ASRM can range from 0 to 20. The total score is obtained by summing up all items and higher scores are indicative of more manic symptomatology. The ASRM revealed good test‐retest reliability (Altman et al., 1997). Cronbach’s alpha in the current study was .78.
*Positive emotion regulation* was assessed with the 17‐item Responses to Positive Affect questionnaire (RPA; Feldman et al., 2008). This questionnaire assesses the positive emotion regulation strategies dampening and positive rumination on three different subscales: (1) dampening (e.g. ‘I don’t deserve this’), (2) self‐focused positive rumination (e.g. ‘I am achieving everything’), and (3) emotion‐focused positive rumination (e.g. ‘Savour this moment’). Respondents were asked to rate how frequently they have these cognitions when experiencing positive emotions on a Likert scale ranging from 1 (almost never) to 4 (almost always). Higher scores indicate more dampening and positive rumination, respectively. For the current study, one item of the dampening subscale (‘This is too good to be true’) was removed from the analyses. This is in line with two previous studies (Kraiss, ten Klooster, et al., 2019; Nelis et al., 2016) that found that this item shows low factor loadings. In the current study, the scores for the dampening subscale can range from 7 to 28, for self‐focused positive rumination from 4 to 16, and for emotion‐focused positive rumination from 5 to 20. The Dutch RPA has shown satisfying psychometric properties in patients with BD (Kraiss, ten Klooster, et al., 2019). Alpha in the current study was .79 for the emotion‐focused and self‐focused subscales and .83 for the dampening subscale.

### Statistical analyses

All statistical analyses were conducted in R (R Core Team, 2018; RStudio Team, 2020). Missing values (3.2%) were handled using random forest imputation using the MissForest package (Stekhoven & Bühlmann, 2012). This imputation method adequately deals with mixed‐type data and is superior to other common imputation techniques in terms of imputation error and maintenance of predictive ability (Waljee et al., 2013). The number of missing values ranged from 1 to 18 for different variables. Categorical variables were dummy coded. The original dataset (*N* = 209) was then split into a training (*n* = 149) and a test set (*n* = 60) using the caret package (Kuhn, 2020).

Three blocks of variables were considered for the multiple regression model. In the first block, the sociodemographic variables age, gender, education, work status and marital status were included. In the second model, the two variables satisfaction and difficulties with social roles were considered (S‐SRPQ). In the third block, medication and admission to a psychiatric hospital, anxiety (HADS‐A) and manic symptoms (ASRM), self‐focused and emotion‐focused positive rumination and dampening of positive emotions (RPA) were additionally considered for the multiple regression model. These potential predictors had to pass three preselection criteria to be included in the multiple regression model. First, categorical variables without sufficient variability (> 80% in one response category) were excluded (Kuhn & Johnson, 2013). Second, simple univariate regression analyses were conducted for each predictor and the criterion personal recovery. Predictors with *p* > .10 were excluded. Third, Pearson intercorrelations between potential predictors were examined to avoid multicollinearity. In case of *r* > .70 or *r* < −.70 between two predictors, the variable with the weaker correlation with the QPR was excluded.

To examine whether combining the two datasets introduced bias, we created a dummy variable indicating to which dataset a participant belongs. Regression models were run to check whether this dummy interacts with any of the variables in explaining personal recovery.

All predictors that passed these criteria were included in the block‐wise multiple regression analyses with personal recovery measured with the QPR as outcome variable. The first model contained sociodemographic variables only, while the second model contained social participation variables. In the third model, psychopathology and emotion regulation variables were entered. To check for significant outliers, Cook‐distances were examined. Cook‐distances > 1 were considered as noteworthy and would require further investigation (Cook & Weisberg, 1982). The variance inflation factor (VIF) was calculated to check for multicollinearity. Values that exceeded 5 were seen as problematic (James, Witten, Hastie, & Tibshirani, 2013). To visually check linearity and homoscedasticity of residuals, normal Q‐Q plots, residuals versus fitted and residuals versus leverage plots were examined and histograms of residuals were plotted and inspected. To statistically test for normal distribution of residuals, Shapiro–Wilk test was conducted. A non‐significant Shapiro–Wilk test is indicative for a normal distribution of the residuals. To compare the fit of the different models, the root‐mean‐square‐error (RMSE) and mean absolute error (MAE) were calculated. Lower RMSE and MAE values are indicative of better model fit.

To create a parsimonious model that comprises fewest variables as possible without compromising predictive ability, we used the train function from the caret package with 10‐fold cross‐validation. The caret package contains functions for regression and classification training. The train function tests different combinations of variables for each possible size of a model and finds the combination of predictors that minimizes the error (Kuhn, 2020). This parsimonious fourth model was then compared with the more complex third model using F‐statistics to determine whether it performs comparably well in explaining variance in personal recovery. To further check whether combining the two datasets leads to confounded findings, we conducted a sensitivity analysis by using the final parsimonious model on both datasets (survey and RCT) separately and by examining whether the explained variance considerably differed in both datasets. Afterwards, the parsimonious model was cross‐validated to test the accuracy of the model. For this, the unstandardized regression coefficients from the final parsimonious model were used to calculate predicted values for the QPR in the test sample. The predicted values from the test sample were then regressed on the observed values from the test sample to examine the accuracy of the predicted values. A β‐coefficient >.70 was considered a strong association and indicative for high model accuracy. To further evaluate the final model, the *R* ^2^ of the test sample was inspected and examined whether it falls within the 95% confidence interval of the *R* ^2^ from the training sample. If the *R* ^2^ fell within the 95% confidence interval, this was considered indicative of a comparable performance of the model in the test sample.

## Results

### Sample characteristics

Table 1 summarizes the sample characteristics. Mean age of the 149 respondents in the training sample was 50.2 (*SD* = 11.4) and about 70% were female. About half of the sample was highly educated (51%) and a relatively high proportion of people was unable to work, had no work or was retired (47.0%). 66 respondents (44.3%) had a diagnosis of BDI, while 70 had a diagnosis of BDII. Almost every respondent (94.6%) took medication because of their BD and almost no one was admitted to a psychiatric hospital in the past 3 months (94.6%).

**Table 1:** Sample characteristics (*N* = 209)

Variable	Category	Training sample (*n* = 149)	Test sample (*n* = 60)
		n (%)	n (%)
Age		*M* = 50.2 (*SD* = 11.36)	*M* = 49.5 (*SD* = 10.47)
Gender	Male	45 (30.2)	11 (18.3)
	Female^c^	104 (69.8)	49 (81.7)
Education	Low	23 (15.4)	6 (10)
	Moderate	50 (33.6)	24 (40)
	High^c^	76 (51)	30 (50)
Work status	Working	79 (53)	33 (55)
	Not working	70 (47)	27 (45)
Marital status	Married or registered relationship^c^	67 (45)	26 (43.3)
	Never been married	42 (28.2)	13 (21.7)
	Divorced	31 (20.8)	14 (23.3)
	Other	9 (6)	7 (11.7)
Diagnosis	BDI^c^	66 (44.3)	30 (50)
	BDII	70 (47.0)	24 (40)
	Other/unknown	13 (8.7)	6 (10)
Currently taking medication^b^	Yes	141 (94.6)	56 (93.3)
	No	8 (5.4)	4 (6.7)
Admitted to psychiatric hospital in the past 3 months^b^	Yes	8 (5.4)	1 (1.7)
	No	141 (94.6)	59 (98.3)

		*M* (*SD*)	*M* (*SD*)
QPR		35.99 (10.55)	35.31 (10.60)
S‐SRPQ difficulties		2.67 (0.83)	2.54 (0.78)
S‐SRPQ satisfaction		2.37 (0.79)	2.57 (0.81)
HADS‐A		8.42 (4.43)	8.54 (4.79)
ASRM		2.17 (2.83)	2.39 (2.79)
RPA emotion‐focused PR		12.11 (3.05)	12.46 (3.04)
RPA self‐focused PR		7.87 (2.84)	7.60 (2.65)
RPA dampening		15.1 (4.71)	15.29 (4.46)

Note

ASRM = Altman Self‐Rating Mania Scale, HADS‐A = Hospital Anxiety and Depression Scale‐Anxiety Subscale, *M* = Mean, PR = Positive Rumination, QPR = Questionnaire About the Process of Recovery, RPA = Responses to Positive Affect Questionnaire, *SD* = Standard Deviation, S‐SRPQ = Short–Social Role Participation Questionnaire.

^a^ Includes participants that were self‐employed, students, housewives or housemen or participants that were doing unpaid voluntary work.

^b^ Variables removed for further analyses because of lack of variance.

^c^ Reference category in regression analyses.

### Simple linear regression analyses

Table 2 summarizes the outcomes of the simple linear regression analysis. From the sociodemographic variables, age was negatively related to personal recovery (β = −.201, *p* < .05). Currently working versus currently not working was positively related to personal recovery (β = .306, *p* < .001). Being divorced versus being in a relationship or being married was negatively associated with personal recovery (β = −.176, *p* < .05). Social participation and psychological variables were all significantly related to personal recovery. Intercorrelations between predictors did not exceed .70 for any of the variables.

**Table 2:** Outcomes of simple univariate regression analyses between predictors and the criterion variable personal recovery

Block	Predictor	*b*	*SE*	*β*	*F*‐value	*p*
1	Age	**−0.187**	**0.075**	**−.201**	**6.19**	.**014**
	Gender_Female_	1.478	1.885	.065	0.62	.434
	Education_Low_	−0.081	2.401	−.003	0.00	.973
	Education_Moderate_	−2.739	1.823	−.123	2.26	.135
	Work status_Working_	**6.439**	**1.655**	.**306**	**15.14**	.**000**
	Marital status_Never married_	**4.671**	**1.889**	.**200**	**6.12**	.**015**
	Marital status_Divorced_	−**4.568**	**2.103**	−**.176**	**4.72**	.**031**
	Marital status_Other_	0.602	3.640	.014	0.00	.869
2	Difficulties with social roles	−**6.762**	**0.892**	−**.530**	**57.51**	.**000**
	Satisfaction with social roles	**8.541**	**0.852**	.**637**	**100.6**	.**000**
3	Anxiety symptoms	−**1.202**	**0.169**	−**.505**	**50.4**	.**000**
	Manic symptoms	**0.907**	**0.298**	.**243**	**9.24**	.**003**
	Self‐focused positive rumination	**1.165**	**0.292**	.**313**	**15.98**	.**000**
	Emotion‐focused positive rumination	**1.369**	**0.262**	.**395**	**27.21**	.**000**
	Dampening of positive emotions	−**0.701**	**0.176**	−**.313**	**15.95**	.**000**

Note

Outcomes printed in bold were considered for multiple regression analyses.

### Interaction effects

No significant interaction was found for any of the predictors and type of diagnosis or gender, suggesting that the relationship between predictors and personal recovery was independent of type of diagnosis and gender. No significant interaction effects were found for the dataset dummy variable, suggesting that the effect of the independent variables did not depend on the dataset. The following interaction terms were found for the interaction with the dataset variable: satisfaction with social roles (*b* = −1.28, *p* = .45), difficulties with social roles (*b* = −1.96, *p* = .27), anxiety symptoms (*b* = −0.13, *p* = .70), manic symptoms (*b* = −0.80, *p* = .39), emotion‐focused positive rumination (*b* = −0.53, *p* = .31), self‐focused positive rumination (*b* = −0.18, *p* = .77), and dampening (*b* = 0.55, *p* = .13).

### Multiple regression analyses

The model summary for the block‐wise multiple regression analyses can be found in Table 3. Variables in the first model explained 13.1% variance in personal recovery, while the second model explained 32.1% additional variance. The third model explained 15.3% additional variance compared to the second model.

**Table 3:** Model summary

Model	*R* ^2^	95% CI	*R*^2^ adjusted	RMSE	MAE	Δ*R* ^2^	F change	Sig. F change
1	.131	0.034 to 0.228	.107	0.654	0.537	.131	5.42	.000
2	.452	0.340 to 0.564	.429	0.519	0.403	.321	41.61	.000
3	.605	0.515 to 0.695	.573	0.441	0.352	.153	10.62	.000
4	.573	0.474 to 0.672	.561	0.458	0.365	.032	1.59	.144

Note

Model 1 = Sociodemographic variables, Model 2 = Sociodemographic + Social variables, Model 3 = Sociodemographic + Social + Psychological variables, Model 4 = Parsimonious model after model training was applied.

Findings show that in the first model, a significant independent predictor of personal recovery was if a participant was currently working. In the third model, satisfaction with social roles and manic symptoms were positively related to personal recovery. VIF scores ranged from 1.050 to 2.592. Cook distances for the third model did not indicate that there were noteworthy outliers that would require further investigation. Shapiro–Wilk test indicated a normal distribution of the residuals (*W* = 0.99, *p* = 0.76).

### Building a parsimonious model

In order to create a parsimonious model based on the third model, we used the train function from the caret package to determine the combination of variables that represent the best fit to the data with the fewest number of variables. This model contained the four variables satisfaction with social roles (β = .442, *p* < .001), anxiety symptoms (β = −.300, *p* < .001), manic symptoms (β = .222, *p* < .001), and emotion‐focused positive rumination (β = .223, *p* < .001). The parsimonious fourth model explained 57.3% variance in personal recovery. The RMSE was 0.458 and the MAE was 0.365. Comparing the more complex third model with the parsimonious fourth model showed that the parsimonious model did not perform significantly worse in predicting personal recovery (Δ*R* ^2^ = .032, *F*_change_(7, 137) = 1.59, *p* = .144). Using the final parsimonious model on both datasets separately, showed a similar variance explained in both datasets (adj. *R* ^2^ = 0.54 in the survey data and adj. *R* ^2^ = 0.57 in the RCT data). This indicates that the final model performed comparably well in both datasets (Table 4).

**Table 4:** Multiple regression analyses with personal recovery as criterion

Model	Predictor	*b*	*SE*	*β*	*t*	*p*
1	Age	−0.088	0.078	−.095	−1.13	.261
	Work status_Working_	5.178	1.742	.246	2.97	.003
	Marital status_NeverMarried_	2.604	1.999	0.111	1.30	.195
	Marital status_Divorced_	−2.128	2.167	.111	−0.98	.328
2	Age	−0.030	0.063	−.032	−0.47	.640
	Work status_Working_	0.424	1.494	.020	0.28	.777
	Marital status_NeverMarried_	3.692	1.611	.158	2.29	.023
	Marital status_Divorced_	0.391	1.777	.015	0.22	.826
	Difficulties with social roles	−1.821	1.223	−.143	−1.62	.107
	Satisfaction with social roles	7.039	1.185	.525	5.94	.000
3	Age	0.013	0.056	.014	0.24	.811
	Work status_Working_	−0.085	1.338	−.004	−0.06	.949
	Marital status_NeverMarried_	2.401	1.449	.103	1.66	.100
	Marital status_Divorced_	0.797	1.550	.031	0.52	.608
	Difficulties with social roles	−1.039	1.057	−.081	−0.98	.327
	Satisfaction with social roles	5.278	1.058	.394	4.99	.000
	Anxiety symptoms	−0.597	0.178	−.251	−3.35	.001
	Manic symptoms	0.853	0.225	.229	3.79	.004
	Self‐focused positive rumination	0.337	0.322	.091	1.05	.296
	Emotion‐focused positive rumination	0.503	0.273	.145	1.85	.067
	Dampening of positive emotions	−0.226	0.137	−.101	−1.65	.101
4	Satisfaction with social roles	5.917	0.847	.442	6.99	.000
	Anxiety Symptoms	−0.704	0.148	−.296	−4.75	.000
	Manic symptoms	0.829	0.208	.222	3.98	.000
	Emotion‐focused positive rumination	0.773	0.197	.223	3.92	.000

### Cross‐validation

**Figure 1:**
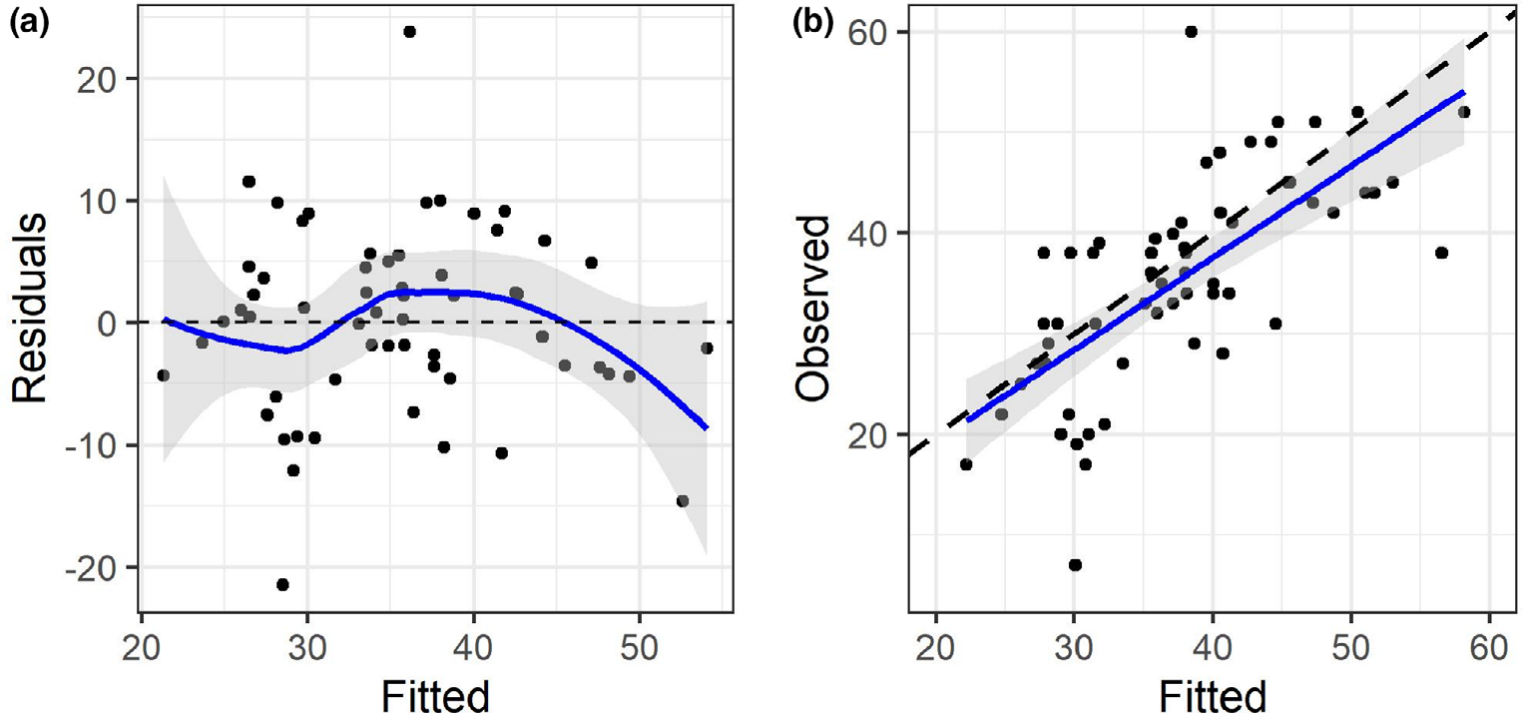
Distribution of error terms (plot A) and relationship between predicted and observed personal recovery scores in the test sample (plot B).

To test the model accuracy of the parsimonious model, we used the regression equation of this model to predict personal recovery in the test sample. We then regressed the predicted values on the observed values from the test sample. Visual inspection of Figure 1 suggests a symmetric distribution of error terms around zero (Plot A) and a relatively strong linear relationship between the predicted and observed values (Plot B). The correlation between predicted and observed values was high (*b* = 0.913, β = .707, *t* = 7.62, *p* < .001), suggesting high model accuracy. The fitted values explained 50% variance in the observed values (*R* ^2^ = .500, 95% CI = 0.340 to 0.660, adj. *R* ^2^ = .491). The explained variance was somewhat lower than in the training sample, but the estimate fell within the 95% confidence interval of the *R* ^2^ estimate in the training sample, indicating that the final model performed comparably well in the independent cross‐validation sample. The RMSE of the model in the test sample was 0.520 and the MAE was 0.397, which was only marginally higher than in the training sample.

## Discussion

The aim of the current study was to explore whether several sociodemographic, social and psychological factors relate to personal recovery in BD. Four variables were identified as most relevantly associated with personal recovery, including satisfaction with social roles, anxiety symptoms, manic symptoms, and emotion‐focused positive rumination. We found that our final parsimonious model with these four variables explained a considerable amount of variance in personal recovery and did not perform worse than the more complex model. This was confirmed in an independent test sample using cross‐validation.

### Main findings

The degree to which an individual was satisfied with the performance of social roles was the strongest correlate of personal recovery and was even stronger related to personal recovery than anxiety symptoms. Prior studies found that satisfaction with social roles is associated with an array of psychological‐ and health‐related outcomes in medical groups (Gignac et al., 2013) as well as with personal recovery and symptomatology in people with schizophrenia (Giusti et al., 2015). The current findings are relevant, as they further solidify the importance of social role participation and suggest that it is an important focus when striving for personal recovery in BD. Social participation might therefore also be a relevant treatment target in this group, for example by specifically aiming at the ability to work or being able to have intimate relationships. This might increase feelings of relatedness and meaning in life, which are seen as two of the main components of personal recovery (Leamy et al., 2011). Interventions that have been shown to improve social role functioning in BD are collaborative care (van der Voort, van Meijel, Hoogendoorn, et al., 2015) and cognitive therapy (Lam, Hayward, Watkins, Wright, & Sham, 2005). Although not yet evaluated in BD, other possible treatment alternatives to improve these outcomes might be vocational rehabilitation (Twamley, Jeste, & Lehman, 2003) or supported employment (Modini et al., 2016).

One other relevant factor that was included in our final model was anxiety symptoms. This finding coincides with previous studies that similarly found significant negative associations between symptomatology and personal recovery in BD (Dodd et al., 2017; Jones et al., 2013). Although clinical recovery is generally considered as being distinct from personal recovery (Best, Law, Pyle, & Morrison, 2020; Macpherson et al., 2016), our findings suggest that anxiety symptoms were still independently associated with personal recovery. Therefore, residual anxiety symptoms might also be a relevant focus of treatment when aiming to improve personal recovery. This might help patients to lead a good and meaningful life by reducing anxiety as possible obstacles for personal recovery. In this context, it is interesting that dampening did not remain a significant correlate in the final model, although we assumed that it is a relevant mechanism for recovery in BD. This finding might be explained by the fact that the bivariate correlation between dampening and anxiety symptoms was significant. Therefore, it is likely that dampening could not independently explain variance in personal recovery beyond anxiety symptoms. This is also relevant for clinical practice, as it indicates that reducing anxiety symptoms might decrease dampening as they seem to overlap. However, the nature of the current data does not allow conclusions about causality, so this remains purely speculative.

Surprisingly, self‐reported manic symptoms were positively related to personal recovery. Previous studies did not report a significant association between manic symptoms and recovery (Dodd et al., 2017; Jones et al., 2013). One possible explanation might be that up until a certain level the feelings and cognitions that are associated with (hypo)manic phases are actually positive. Thus, an individual might feel good, energized and may experience inflated self‐confidence, which might also result in a relatively high score in personal recovery. For therapists and patients, our findings suggest that they may not need to be too concerned of positive moods per se and that improving these positive aspects is also relevant when striving for recovery. Actually, feelings similar to hypomanic experiences are reported by the majority of people in the general population (Jones, Mansell, & Waller, 2006; Udachina & Mansell, 2007), suggesting that positive mood itself may not always be the main problem in BD. This corresponds with Mansell (2016), who argues that positive moods should be accepted and cherished in BD, but that it is also important to distinguish between the destructive and constructive power of positive mood. A qualitative study by Russell and Moss (2013) shows that it is possible for people with BD to develop this insight, which may be an important step towards recovery. Nonetheless, it should also be mentioned that (hypo)manic episodes can have devastating effects. This might make it difficult for patients, therapists and their environment to impartially accept and cherish positive feelings and find the right balance between fostering and controlling positive feelings. It is also important to mention that the present study predominantly captured patients with subsyndromal manic symptomatology. This is indicated by the mean ASRM score in the current study, which was relatively low and comparable to previous studies that included euthymic patients with BD (van der Voort, van Meijel, Goossens, et al., 2015; Zyto, Jabben, Schulte, Regeer, & Kupka, 2016). Furthermore, full‐blown manic episodes are relatively rare and the course of BD is usually dominated by depressive episodes and euthymic states (Judd et al., 2002, 2003; Kupka et al., 2007). This makes it impossible to make inferences regarding the relationship between full‐blown manic symptoms and personal recovery in the current study.

Emotion‐focused positive rumination was also included as relevant predictor in the final model. Research regarding the role of positive rumination for personal recovery in BD remains absent and to our knowledge, this was the first study to explore the association between positive emotion regulation and personal recovery in BD. Our findings are in line with prior studies in community and college samples that similarly found that positive rumination is positively related to recovery‐related outcomes, such as self‐esteem (Feldman et al., 2008) and life satisfaction (Quoidbach et al., 2010) and negatively related to depressive symptoms (Nelis et al., 2016). Our findings further solidify the relevance of positive rumination and generalize the relevance of this process to the concept of personal recovery in BD. This finding is also relevant for clinical practice, since positive rumination might be trained by practicing with savouring strategies (Quoidbach et al., 2010). Some examples of savouring strategies that have been shown to be related to increased experience of positive emotions include capitalizing (i.e. communicating and celebrating positive events) (Langston, 1994) and mental time travel (i.e. vividly remembering positive events) (Suddendorf & Corballis, 2007).

Sociodemographic variables explained a relatively small amount of variance in personal recovery, suggesting that personal recovery is reachable independent from gender, age and education. Interestingly, whether someone was working had an independent positive impact on personal recovery in the first model. This indicates that employment status contributes to personal recovery, which corresponds to previous research that found that employment status was significantly associated with personal recovery in BD, even after controlling for other clinical outcomes (Dodd et al., 2017). Working status was not a significant predictor anymore after including social role participation in the model. This was most likely because the social role participation questionnaire also includes items about whether the respondent is able to work and therefore actual employment status could not explain much additional variance beyond perceived social role participation. It is also important to mention that people were also classified as working in the current study if they had unpaid voluntary work. This implies that also having unpaid work might already be beneficial for recovery.

### Implications

Our findings show that social role participation as well as anxiety symptoms and positive rumination appear to be relevant predictors for personal recovery in BD. Focussing on these outcomes during treatment might help to improve recovery. Therapies such as Cognitive Behavioural Therapy or Acceptance and Commitment Therapy may be beneficial to cope with anxiety. Collaborative care (van der Voort, van Meijel, Hoogendoorn, et al., 2015), vocational therapy (Twamley et al., 2003), or supported employment (Modini et al., 2016) has been found to be effective for social functioning and functional recovery. Exercising savouring strategies and positive psychology interventions might represent promising ways to cultivate positive emotions and increase positive rumination. Also, a positive psychology intervention has recently been shown be effective in improving recovery‐related outcomes such as optimism in a randomized controlled pilot trial in people with BD (Celano et al., 2020). Combining these interventions might help to comprehensively increase recovery in BD. Nonetheless, it is also desirable that future research further investigates the effectiveness of novel forms of psychotherapy to improve recovery (Murray et al., 2017), including third‐wave therapies or positive psychotherapy.

### Limitations

The current study has several limitations. First, the data were cross‐sectional. Therefore, no conclusions about causality and direction of effect can be drawn. Second, about one half of the sample did not have a confirmed diagnosis of BD, as these participants were gathered via convenience sampling for an online survey. However, they were gathered through the Dutch patient association for people with BD and were asked to self‐report their diagnosis. Therefore, it can be assumed that a great part of the sample actually had BD. Third, our study only included a specific selected set of predictors. Therefore, no complete overview of all possible factors that might contribute to personal recovery can be given. For example, we merely included anxiety symptoms as predictor, but not depressive symptoms. The reason for this is that depression was measured with different scales in the two datasets, making it impossible to compare depression scores of participants between the two datasets. Considering that depression might also be present in remission (Vieta, Sanchez‐Moreno, Lahuerta, & Zaragoza, 2008) and that there is a link between depression and recurrence (Pinto et al., 2020), depression may also be related to personal recovery and we may thus miss an important factor contributing to personal recovery in the current study.

### Conclusion

The current findings suggest that psychopathology as well as social role participation and positive rumination are important independent predictors for personal recovery in BD. These insights widen the knowledge of what contributes to personal recovery in this specific group. The results also have implications for clinical practice, as including these factors into treatment might improve personal recovery in people with BD. We encourage future research to further explore predictors of personal recovery in BD and examine which interventions effectively enhance personal recovery. This might lead to more effective recovery‐based treatments and help to enhance recovery beyond clinical and functional recovery.

## Conflicts of interest

The authors declare that there is no conflict of interest regarding the publication of this article.

## Data Availability

The data that support the findings of this study are available from the corresponding author upon reasonable request. Data of the randomized controlled trial will not be available before completion of the trial.
